# Editorial: Beyond PD-1: novel checkpoint receptors and ligands as targets for immunotherapy

**DOI:** 10.3389/fimmu.2025.1563383

**Published:** 2025-02-11

**Authors:** Jesse Haramati, Fabiola Solorzano-Ibarra, Dallas B. Flies

**Affiliations:** ^1^ Laboratorio de Inmunología Traslacional, Departamento de Biología Celular y Molecular, Centro Universitario de Ciencias Biológicas y Agropecuarias, Universidad de Guadalajara, Zapopan, Jalisco, Mexico; ^2^ Estancias Posdoctorales por México, Consejo Nacional de Humanidades, Ciencias y Tecnologías (CONACHYT), México City, Mexico; ^3^ Instituto en Investigación en Enfermedades Crónico Degenerativas, Centro Universitario de Ciencias de la Salud, Universidad de Guadalajara, Guadalajara, Jalisco, Mexico; ^4^ NextCure, Inc., Beltsville, MD, United States

**Keywords:** ICI Immunotherapy, cancer, tumor, personalized & precision medicine, checkpoint blockade (ICB) therapy

In recent years immunotherapy has become one of the most promising technologies to treat advanced tumors. The idea of modulating the immune system has improved the treatment of cancer patients. While great success has been seen with inhibitors of PD-1, the potential for targeting other cells and ligands in the tumor microenvironment remains vast. This Research Topic emphasizes the potential of targeting both inhibitory and co-stimulatory receptors to reinvigorate anti-tumor immune responses, mainly focusing on receptors other than PD-1.

Some of the most promising research concerns the potential of targeting VISTA, an inhibitory B7 family molecule that can be expressed on tumor cells and myeloid cells in the tumor microenvironment, particularly in immunologically cold tumors. Duan et al. explored the role of VISTA in acute myeloid leukemia and multiple myeloma. VISTA co-expresses with other checkpoint receptors (e.g., PD-1, TIM-3) and may influence prognosis, being associated with poor overall survival in pancreatic or prostate cancers, making it a potential target for immunotherapy. Iadonato et al. report on the creation of a humanized anti VISTA antibody that was tested in colon cancer and melanoma models, followed by promising toxicology and pharmacokinetics studies in monkeys.

TIM-3 and LAG-3 are inhibitory immune checkpoints implicated in immune modulation that have received growing interest in recent years. In ovarian cancer, TIM-3 affects the immune microenvironment and T-cell function. Chang et al. assessed its potential as a therapeutic target, discussing both the regulatory role of TIM-3 and the challenges in developing effective TIM-3-based therapies. Luo et al. explored LAG-3 and the promising status of several inhibitors under development, while Amrane et al. investigated the potential for HLA-DR, the ligand for LAG-3. While its role in hot tumors may be important, HLA-DR’s potential for direct clinical targeting is limited by its expression on almost all antigen presenting cells and the resulting adverse effects that would occur in the case of blockade.

Likewise, CD5 is another inhibitory checkpoint that shows potential. Blocking CD5, an immune checkpoint receptor on T cells, enhances T-cell-mediated anti-tumor immunity. In a mouse model of poorly immunogenic breast cancer, Alotaibi et al. showed that anti-CD5 treatment increased CD8+ T-cell activation, delayed tumor growth, and improved anti-tumor responses. CD5 blockade may be a promising new therapeutic strategy for enhancing immunity against solid tumors.

While this Research Topic focused on expanding the gamut of targets beyond the PD-1/PD-L1 axis, it is interesting to consider PD-L2, due to its high affinity for PD-1, as an alternative or complementary target. Yang et al. reviewed PD-L2’s role in immune evasion and its expression in various tumors, suggesting that targeting PD-L2 might complement current PD-L1/PD-1 therapies and help refine patient selection for immunotherapy. Notably, microbiota regulate the PD-1 pathway, and this opens the door to focusing on characterizing the different intestinal populations that might make a patient more likely to express PD-L2/L1 and respond positively to immunotherapy. The discovery of new pathways such as PD-L2 binding to RGMb on CD8+ T cells has also expanded the importance of PD-L2. However, clinical trials for antibodies targeting PD-L2 remain limited, but there have been recent studies focused on bispecific anti PD-1/2 constructs, and small molecule inhibitors of PD-L2, and these avenues bear watching in the near future.

Moving past inhibitory checkpoints, it is important to consider other molecules, either as targets themselves or markers of an exhaustive state that could be prognostic. Reolo et al. found that CD38, originally considered a marker of activated cells, was a key marker of exhausted T cells in hepatic cell carcinoma, and a potential target as well.

One pathway that has yet to be fully explored is the role of inhibitory receptors in plasmacytoid dendritic cells. Plasmacytoid dendritic cells, a subset of dendritic cells characterized by the ability to secrete massive amounts of type-I interferons, play a critical role in immune responses, but in tumors, they are often suppressed via inhibitory molecules and receptors. Tiberio et al. reviewed this, focusing on prostaglandin E2, TGFβ, and IL-10 produced by tumor cells and inhibitory receptors including BDCA-2, ILT7, NKp44, DCIR, ILT2, LAIR1, TIM-3, CD300c and CD300a that can be hijacked by tumor cells, thus hindering pDC activation. Targeting these receptors could enhance pDC-mediated immunity and improve cancer immunotherapy outcomes.

Finally, the extracelular matrix of tumors plays a critical role in immune regulation, often inhibiting immune cell infiltration. The altered ECM in tumors can suppress immune responses, reducing the effectiveness of immunotherapies like ICIs. Understanding how ECM components, particularly collagens, modulate tumor immunity could lead to novel strategies for overcoming immune suppression in the tumor microenvironment. Flies et al. explored the literature on this question, showing the potential of inhibiting immunosuppressive TGF-β or LAIR-1 or targeting immune modulating cytokines directly to the TME using molecules that specifically target the dysfunctional ECM.

This Research Topic explores the role of alternative activating and inhibitory immune checkpoints as regulators and therapeutic targets in the tumor microenvironment from different types of cancer. We believe that the continued investigation into novel cellular targets and the integration of both inhibitory and co-stimulatory pathways opens avenues for innovative therapies, including multi-specific redirectors and functionalized CAR-T or NK cells. New research should focus on novel targets and the combination of these with conventional treatments like first generation (PD-1/CTLA-4) immunotherapy and chemotherapy; currently VISTA is one of the most promising of these targets. We thank all the authors and reviewers for their contributions to this Research Topic.

